# Markedly Increased Diamine Oxidase During Acute Anaphylaxis Is Associated With an Underlying Clonal Mast Cell Disorder

**DOI:** 10.1111/cea.70227

**Published:** 2026-01-29

**Authors:** Matija Rijavec, Žan Kogovšek, Jezerka Inkret, Peter Kopač, Mitja Košnik, Peter Korošec

**Affiliations:** ^1^ University Clinic of Respiratory and Allergic Diseases Golnik Golnik Slovenia; ^2^ Biotechnical Faculty University of Ljubljana Ljubljana Slovenia; ^3^ Faculty of Medicine University of Maribor Maribor Slovenia; ^4^ Institute of Forensic Medicine, Faculty of Medicine University of Ljubljana Ljubljana Slovenia; ^5^ Faculty of Medicine University of Ljubljana Ljubljana Slovenia; ^6^ Faculty of Pharmacy University of Ljubljana Ljubljana Slovenia

**Keywords:** anaphylaxis, clonal mast cell disorder, diamine oxidase (DAO), *KIT* p.D816V, tryptase

## Abstract

**Introduction:**

Diamine oxidase (DAO) degrades histamine, the key mediator in anaphylaxis, yet its relationship with clonal mast cell disorder (CMD) in the context of anaphylaxis is unclear. We evaluated whether DAO during anaphylaxis differs by CMD status.

**Methods:**

We enrolled 35 emergency‐department patients with acute anaphylaxis to drugs (7 patients), food (2 patients), or *Hymenoptera* venom (26 patients). Tryptase, DAO, and histamine degradation were measured during anaphylaxis and convalescence. CMD was defined by detecting *KIT* p.D816V in peripheral blood leukocytes using highly sensitive qPCR. Post‐mortem DAO and tryptase were also compared in two fatal *Hymenoptera* venom‐triggered anaphylaxis (HVA) cases with CMD versus 13 non‐anaphylaxis controls.

**Results:**

*KIT* p.D816V was detected in 6 (17%); all had severe HVA and normal basal tryptase. During anaphylaxis, DAO increased markedly in CMD (median 1142%), but only modestly in *KIT* p.D816V‐negative patients (median 20%; *p <* 0.0001), independent of trigger or severity. Acute DAO was ~5‐fold higher in CMD (median 101 vs. 18 U/mL), while convalescent DAO was similar (both 14 U/mL). Despite markedly elevated DAO, we observed impaired histamine degradation in acute anaphylaxis plasma. Receiver‐operating‐characteristic analyses showed strong discrimination for CMD using acute DAO (AUC 0.92; cut‐off 53 U/mL; sensitivity 83%; specificity 97%) and percentage increase from convalescence (AUC 0.97; cut‐off 223%; sensitivity 83%; specificity 100%). Post‐mortem DAO lacked specificity, whereas post‐mortem tryptase supported the diagnosis of fatal anaphylaxis and CMD.

**Conclusion:**

DAO concentrations rise markedly during anaphylaxis in CMD and may help identify individuals at the highest risk. Further studies should refine the diagnostic utility and elucidate the mechanisms by which DAO may amplify anaphylaxis in CMD.

## Key Messages

- Massive DAO release during severe anaphylaxis is restricted to clonal mast cell disorders.
- Possible functional support for the reported association between *KIT* p.D816 and anaphylaxis severity.
- Measuring DAO during acute anaphylaxis may help identify individuals at the highest risk.

## Graphical abstract

We provide the first evidence that pronounced release of DAO during anaphylaxis is restricted to patients with clonal mast cell disorder. This finding offers possible functional support for the reported association between *KIT* p.D816V and anaphylaxis severity. Measuring DAO during acute anaphylaxis may help identify individuals at the highest risk.

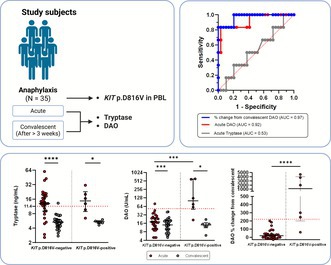

## 1 Introduction

Anaphylaxis is a severe, systemic, potentially life‐threatening allergic reaction that occurs rapidly after exposure to an allergen [1, 2]. It is triggered mainly by food, drugs, or insect stings and characterised by the abrupt release of mediators from mast cells and basophils, with histamine being one of the most critical mediators implicated in the pathophysiology of this condition [1, 2]. Clonal mast cell disorders (CMD) are associated with severe anaphylaxis, especially *Hymenoptera* venom‐triggered anaphylaxis (HVA) [3, 4, 5, 6, 7]. Recent studies have highlighted the importance of testing for *KIT* p.D816V variant in peripheral blood leukocytes (PBL) in patients with anaphylaxis as a screening tool for underlying CMD [5, 8, 9]. The presence of an activating variant in *KIT*, most often being *KIT* p.D816V, in PBL represents one of the minor criteria for the diagnosis of systemic mastocytosis and is thus highly indicative of CMD, most often bone marrow mastocytosis, monoclonal mast cell activation syndrome and indolent systemic mastocytosis [5, 9]. How expansion of a KIT D816V^+^ cell clone modifies the severity of anaphylaxis requires additional investigation since the “releasability” of MCs in the context of anaphylaxis depends on several factors, including the burden of involved MCs and the cellular activation level, based on intrinsic (KIT D816V‐related) MC characteristics [10]. Previous studies have shown that recombinant human stem cell factor (KIT ligand) promotes human MC functional activation in vivo and targeting KIT reduces MC number and reactivity [11, 12]. A better understanding of the functional behaviour of KIT D816V‐positive cells will provide important insight into the underlying mechanism of anaphylaxis and its long‐term management [10].

While typical treatments focus on the administration of epinephrine [1, 2], understanding the role of enzymes like diamine oxidase (DAO) in degrading histamine offers insights into potential therapeutic strategies and the biological mechanisms underlying anaphylaxis. DAO is one of the two fundamental enzymes in humans, the other being histamine‐N‐methyltransferase, involved in the catabolic pathways of histamine through the oxidative deamination of histamine and other biogenic amines [13, 14]. It is mainly expressed in epithelial cells of the small intestine, the placenta, the kidneys, and the liver, stored in vesicular structures, and released into the bloodstream after stimulation [14, 15]. In normal physiological conditions, DAO rapidly degrades extracellular histamine, which is ingested or released during a reaction, acting as a preventive measure from excess histamine accumulating in the body and preventing allergy‐like histamine excess symptoms [13, 16]. The concentration and activity of DAO can vary among individuals, influenced by genetic factors, dietary intake, and the presence of certain medical conditions, thus playing a significant role in an individual's response to histamine exposure [13, 14, 17]. Reduced DAO activity leads to decreased intestinal degradation of ingested histamine, causing histamine accumulation and histamine intolerance (HIT) [13, 14, 17]. Individuals with low DAO activity may experience symptoms similar to anaphylaxis when consuming foods high in histamine, such as fermented products, aged cheeses, and certain alcoholic beverages [13, 14, 17]. Apart from well‐known HIT, altered DAO levels have also been linked to allergic diseases since serum DAO levels were higher in patients with atopic asthma and allergic rhinitis than in controls [18]. DAO also correlated with disease severity [18]. Furthermore, increased DAO activity during acute asthmatic attacks in children was reported [19], and a histamine‐rich diet resulted in more severe symptoms in asthmatic patients during a randomised, two‐period (high‐ or low‐histamine diet) study in children with asthma [20].

Although changes in DAO during anaphylactic events are rarely demonstrated, the evidence presents a moderate to marked increase of histaminolytic activity during severe episodes in rabbits and guinea pigs [21, 22]. Besides, a small study reported the massive release of DAO during anaphylaxis in mastocytosis patients [23], confirming previous animal data of DAO release into the circulation during severe anaphylaxis [21, 22]. A 100‐fold increase compared to the basal state indicates that DAO has great potential to be an important biomarker during anaphylactic episodes [23]. However, the study included only two mastocytosis patients experiencing altogether four severe and one mild anaphylactic event [23]. Besides, it remains unknown whether DAO increases during anaphylaxis also in non‐mastocytosis patients.

To address this gap in our knowledge, we evaluated serum DAO concentrations during acute severe anaphylaxis and in the convalescent state of the patients to determine how DAO concentration changes during severe anaphylaxis, particularly in patients with CMD. Furthermore, we also evaluated whether DAO concentration changes during anaphylaxis are comparable between patients with and without CMD. Additionally, we compared postmortem DAO and tryptase concentrations from two fatal HVA cases with underlying CMD [24] to those from 13 control decedents who died of non‐anaphylactic causes.

## 2 Methods

### 2.1 Study Subject

The patients were recruited during a 2‐year period (2018–2020), presenting with an acute episode of anaphylaxis at the emergency department at the University Clinic of Respiratory and Allergic Diseases Golnik, Slovenia. We retrospectively evaluated 35 (14 female, age range 19–77 years) patients with sufficient samples. Blood samples were collected during the reaction (at presentation to the emergency department), and convalescent samples were collected at least three weeks (median 70 days) after the anaphylactic episode. Tryptase and DAO concentrations were measured from blood samples collected at both time points. The patients included are part of a previous Vantur et al. study [25], and we included all subjects with sufficient samples to determine their DAO concentration and *KIT* p.D816V variant status.

We included two additional cases of fatal HVA with postmortem identified underlying CMD (both males, aged 31 and 71 years), which were previously described in detail [24]. As a control group, we included deaths in which the final autopsy diagnosis was not anaphylaxis (13 in total, 4 female, median 63 years, age range 20–80 years). The causes of death in the control group were cardiac arrest in 4 and injury/external causes in 9 patients (3 car/motorbike accident, 5 suicide by hanging, 1 pneumonia). In all cases, blood samples were taken from the femoral vein, which was properly prepared and punctured during an autopsy performed less than 96 h (median 36 h) after death.

The study was conducted in accordance with the amended Declaration of Helsinki. Ethical approval was obtained from the Slovenian National Medical Ethics Committee (Approval numbers 150/09/13, 0120‐424/2020‐3, and 0120‐180/2025‐2711‐3).

### 2.2 Total Tryptase Testing

Levels of total serum tryptase were measured with a commercially available fluorescence enzyme ImmunoCAP immunoassay (Thermo Fisher Scientific, Uppsala, Sweden) following the manufacturers' instructions. The detection limit of the test is determined as 1 ng/mL, and the normal range for total tryptase levels in serum ranges from 1 to 11.4 ng/mL.

### 2.3 Determination of Serum DAO Concentration

The serum DAO concentration was measured in acute and convalescent samples with enzyme‐linked immunosorbent assay (ELISA). Commercially available IDKDiamineoxidase (DAO) ELISA (Immundiagnostik AG, Germany) was used to perform the tests according to the manufacturers' instructions. Samples with concentrations above the test range were further diluted and re‐assayed till the actual value was obtained, up to 1000 U/mL. Measurements > 1000 U/mL were truncated at 1000 U/mL. All results are presented as U/mL.

### 2.4 Determination of Histamine Degradation in Serum/Plasma

Histamine degradation was assessed using a method adapted from Boehm [23]. Briefly, serum or plasma was incubated with histamine (final concentration 100 ng/mL; Sigma‐Aldrich, St. Louis, MO, USA) for 60 min at 37°C. Reactions were terminated by adding diminazene aceturate to a final concentration of 50 μmol/L (Sigma‐Aldrich), a potent and selective inhibitor of DAO. Samples were stored at −30°C until histamine quantification using a Histamine ELISA kit (ab285333; Abcam, Cambridge, UK), following the manufacturer's instructions. Values > 100 ng/mL were reported as 100 ng/mL, the final concentration of added histamine and the upper limit of the assay. Histamine degradation was determined in 32 paired blood samples (acute and convalescent) for which sufficient serum or plasma was available. Additionally, plasma from a pregnant woman with a high DAO concentration was included as a positive control and measured in triplicate.

### 2.5 *KIT* p.D816V Missense Variant Assay

Genomic DNA from whole blood was isolated with QIAamp DNA blood mini kit (Qiagen, Hilden, Germany). The *KIT* c.2447A>T, p.D816V missense variant was assayed with allele‐specific quantitative PCR as described previously [5, 8].

### 2.6 Statistical Analysis

All the data were statistically analysed using GraphPad Prism (GraphPad Software, Boston, MA, USA). Normality was assessed with the D'Agostino & Pearson test, and then based on the distribution, a paired *t*‐test or a Wilcoxon test was used to determine the statistical difference between paired samples. For comparisons between groups, we used the Mann–Whitney *U* test or unpaired *t*‐test, as appropriate. Determination of a cut‐off value was done with receiver operating characteristic curve (ROC curve) analysis. Numeric data were presented with medians and interquartile ranges (IQR). *p*‐Values below 0.05 were considered statistically significant.

## 3 Results

### 3.1 Clonal Mast Cell Disorders Are Common Among Patients With Severe Sting Anaphylaxis

Anaphylaxis was predominantly triggered by *Hymenoptera* venom (26/35), followed by medication (7/35), and by food in (2/35) patients. Anaphylaxis was graded on Mueller severity scale, we had two patients with grade I, four with grade II, twelve with grade III and 17 with grade IV reaction; of the severe grade IV reactions, 16 were caused by an insect sting and one by medication (Table 1). The *KIT* p.D816V variant was detected in 17% (6/35) of the evaluated patients and 35% (6/17) of patients with severe (Mueller grade IV) anaphylaxis (Table 2). Importantly, all patients with identified CMD (*KIT* p.D816V‐positive patients) experienced severe HVA and had normal basal serum tryptase (BST) levels, ranging from 4.63 to 6.01 ng/mL. One patient with detectable *KIT* p.D816V in PBL had REMA score < 2. The presence of *KIT* p.D816V variant in extracutaneous organs (including PBL) represents one of the minor criteria for the diagnosis of systemic mastocytosis [9, 26], hence bone marrow (BM) examination to classify CMD was performed in three patients (the other three patients rejected BM biopsy), which confirmed BM involvement in all three patients: monoclonal mast cell activation syndrome (MMAS) in two patients and bone marrow mastocytosis (BMM) in one patient according to WHO criteria [9, 26].

**TABLE 1:** Clinical and laboratory characteristics.

Characteristic	Patients with an anaphylaxis (*N* = 35)
Sex, *N* (%)	
Male	21 (60)
Female	14 (40)
Age (years), median (range)	53 (19–77)
Trigger, *N* (%)	
*Hymenoptera* venom	26 (74)
Drugs	7 (20)
Food	2 (6)
Reaction severity grade,^a^ *N* (%)	
I	2 (6)
II	4 (11)
III	12 (34)
IV	17 (49)
REMA score,^b^ *N* (%)	
≥ 2	7 (27)
< 2	19 (73)
Emergency treatment, *N* (%)	
Epinephrine	20 (57)
Corticosteroids	32 (91)
Antihistamines	35 (100)
BST level (ng/mL), median (range)	5.36 (2.74–13.2)
Time from onset of episode to blood collection at the ED, median (range) in minutes	107 (20–1237)
Convalescent sampling time after episode, median (range) in days	70 (24–369)

Abbreviations: BST, basal serum tryptase; ED, emergency department; REMA score, Red Espanola de Mastocitosis score.

^a^ Grades were assigned according to Mueller.

^b^ REMA score was calculated only for *Hymenoptera* venom‐triggered anaphylaxis (*n* = 26).

**TABLE 2:** Clinical and laboratory characteristics in *KIT* p.D816V‐positive and ‐negative patients.

Characteristic	Patients with an anaphylaxis episode (*N* = 35)		*p*
	*KIT* p.D816V‐positive (*N* = 6)	*KIT* p.D816V‐negative (*N* = 29)	
Sex, *N* (%)			
Male	3 (50)	18 (62)	0.66
Female	3 (50)	11 (38)	
Age (years), median (range)	49.5 (34–72)	55 (19–77)	0.67
Trigger, *N* (%)			
*Hymenoptera* venom	6 (100)	20 (69)	0.30
Medication	0 (0)	7 (24)	0.31
Food	0 (0)	2 (7)	> 0.99
Reaction severity grade,^a^ *N* (%)			
I	0 (0)	2 (7)	> 0.99
II	0 (0)	4 (14)	> 0.99
III	0 (0)	12 (41)	0.07
IV	6 (100)	11 (38)	0.**007**
REMA score,^b^ N (%)			
≥ 2	5 (83)	2 (10)	0.**002**
< 2	1 (17)	18 (90)	
Emergency treatment, *N* (%)			
Epinephrine	4 (67)	16 (55)	0.68
Corticosteroids	6 (100)	26 (90)	> 0.99
Antihistamines	6 (100)	29 (100)	> 0.99
BST level (ng/mL), median (range)	5.44 (4.63–6.01)	5.31 (2.74–13.2)	0.88
Time from onset of episode to blood collection at the ED, median (range) in minutes	65 (40–180)	109 (20–1237)	0.11
Convalescent sampling time after episode, median (range) in days	103.5 (39–303)	70 (24–369)	0.62

*Note:* Bold indicates *P* values < 0.05.

Abbreviations: BST, basal serum tryptase; ED, emergency department; Red Espanola de Mastocitosis score.

^a^ Grades were assigned according to Mueller.

^b^ REMA score was calculated only for *Hymenoptera* venom‐triggered anaphylaxis (*n* = 26).

### 3.2 Acute Serum Tryptase Levels Were Comparably Increased in Patients With or Without Underlying Clonal Mast Cell Disorder

**FIGURE 1:**
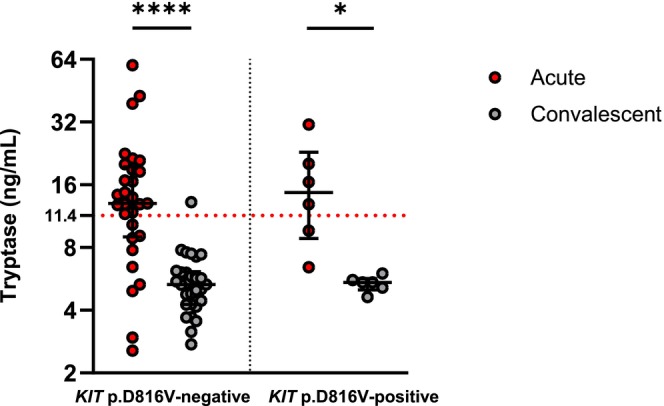
Serum tryptase concentration in patients during acute episodes and in convalescent samples collected later. The dotted line represents currently considered cut‐off (11.4 ng/mL). ****p < 0.0001; *p < 0.05; Wilcoxon matched‐pairs signed rank test.

During the acute phase of anaphylactic reaction, tryptase concentration increased in both designated subgroups of patients, while no significant differences can be shown between the subgroups of *KIT* p.D816V‐positive and ‐negative patients (Figure 1). Importantly, acute serum tryptase was increased (≥ 11.4 ng/mL) in 69% (24/35) of patients while comparing the acute with convalescent sample and using the 20% + 2 tryptase formula (an event‐related transient elevation of the serum tryptase level by at least 20% over the individual baseline plus 2 ng/mL) [27], it was found to be increased in 74% (26/35) of patients. Interestingly, increased acute tryptase using the 20% + 2 formula was confirmed in 85% (22/26) HVA patients and only 44% (4/9) medication‐ or food‐triggered anaphylaxis patients.

### 3.3 Higher Increase of Acute DAO in Patients With Underlying Clonal Mast Cell Disorder

**FIGURE 2:**
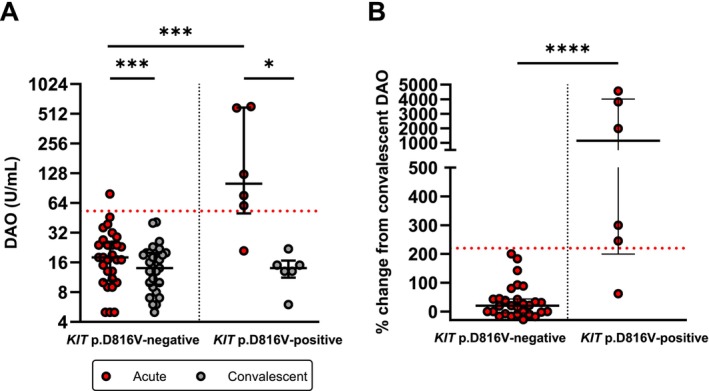
(A) Serum DAO concentration levels in patients during acute episodes and in convalescent samples collected afterwards. The dotted line represents ROC determined cut‐off for discriminating *KIT* p.D816V‐positive patients. ***p < 0.001; *p < 0.05; Wilcoxon matched‐pairs signed rank test. (B) Percentage change of DAO during acute episode compared to convalescent sample. The dotted line represents ROC determined cut‐off for discriminating *KIT* p.D816V‐positive patients. ****p < 0.0001; Mann–Whitney test.

Statistically significant increase in DAO concentration during the anaphylactic reaction was observed, both in *KIT* p.D816V‐positive and *KIT* p.D816V‐negative patients (*p =* 0.0007 and *p =* 0.03; Figure 2A). However, a markedly, approximately 50‐fold higher increase in DAO (median 1142%) was observed in *KIT* p.D816V‐positive individuals, in comparison to *KIT* p.D816V‐negative patients (median 20%) (*p <* 0.0001; Mann–Whitney test; Figure 2B). Furthermore, absolute DAO concentrations during anaphylaxis were significantly higher in *KIT* p.D816V‐positive patients compared to *KIT* p.D816V‐negative patients, median 101 U/mL and 18 U/mL, respectively (*p =* 0.0004; Figure 2).

### 3.4 Impaired Histamine Degradation in Acute Anaphylaxis Plasma Despite Markedly Increased DAO

**FIGURE 3:**
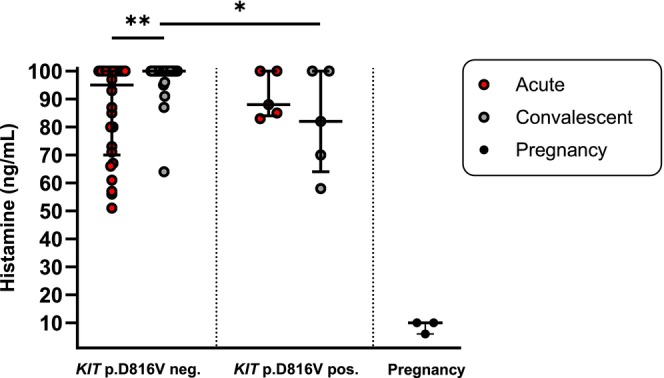
Histamine degradation in serum and plasma after 60 min. Serum or plasma samples were spiked with exogenous histamine (final concentration 100 ng/mL) and incubated for 60 min. Histamine concentrations (ng/mL) measured after incubation are shown as median with interquartile range (IQR). **p < 0.01; Wilcoxon matched‐pairs signed rank test. *p < 0.05; Mann–Whitney test.

Compared with a pregnancy plasma sample with high DAO (518 U/mL), which degraded exogenously added histamine (100 ng/mL) almost completely within 60 min (to 10 ng/mL), patient serum or plasma samples showed limited degradation of histamine. After 60 min of incubation, histamine concentrations did not decrease below 50 ng/mL in any acute anaphylaxis sample (Figure 3), despite DAO concentrations reaching up to 606 U/mL. Notably, the two acute samples with the highest DAO levels (606 and 587 U/mL) showed only modest histamine degradation, with residual histamine concentrations of 100 and 83 ng/mL, respectively, after 60 min. When stratified by *KIT* p.D816V status, greater histamine degradation in acute versus convalescent samples was observed only in *KIT* p.D816V‐negative patients (median histamine after 60 min: 95 vs. 100 ng/mL; *p* = 0.0011; Figure 3). In contrast, no difference between acute and convalescent samples was evident in *KIT* p.D816V‐positive patients, despite these patients exhibiting the highest absolute DAO concentrations during anaphylaxis.

### 3.5 DAO Is Highly Increased in Fatal Anaphylaxis Cases With Underlying Clonal Mast Cell Disorder

We additionally measured DAO in post‐mortem serum from two previously reported fatal HVA cases with underlying clonal mast‐cell disease (CMD) [24]. DAO was markedly elevated in both (> 1000 U/mL), which corroborates our findings in the emergency department study, which demonstrated a highly increased acute DAO in patients during a sting anaphylaxis episode and with underlying CMD. However, DAO showed substantial variability and elevated DAO levels also in the control group (deaths due to cardiac arrest or injury/external causes), ranging from 10 to > 1000 U/mL (median 202 U/mL; IQR 17–> 1000 U/mL), with 5 of 13 controls exceeding 1000 U/mL (*p =* 0.305). Median DAO appeared higher after cardiac arrest (906 U/mL; IQR 210–> 1000 U/mL) than after injury/external causes (86 U/mL; IQR 17–> 1000 U/mL), but this difference was not statistically significant (*p =* 0.663). Collectively, these findings highlight the limited specificity of DAO in the post‐mortem setting.

**FIGURE 4:**
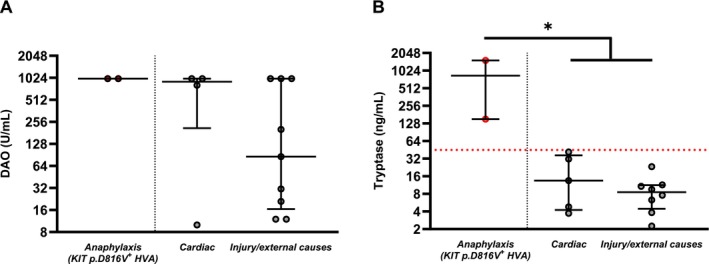
Post mortem serum (A) DAO and (B) tryptase concentrations in anaphylactic deaths and control groups. Dotted lines indicate previously suggested tryptase cut‐off levels of 45 ng/mL [28, 29]. *p < 0.05; Mann–Whitney test.

By contrast, serum tryptase was markedly elevated in both fatal HVA cases with postmortem‐identified underlying CMD cases (1530 and 152 ng/mL), while remaining low in controls (overall median 9.5 ng/mL; IQR 4–18 ng/mL), a difference that reached statistical significance (*p* = 0.019; Figure 4). Tryptase medians were similarly low across the two control subgroups (injury/external causes: 8.6 ng/mL; cardiac arrest: 13.5 ng/mL; *p =* 0.435). These data support post‐mortem tryptase as a useful discriminating biomarker for fatal anaphylaxis in the presence of CMD.

### 3.6 Increase in DAO During Anaphylactic Reaction Is Highly Indicative of an Underlying Clonal Mast Cell Disorder

**FIGURE 5:**
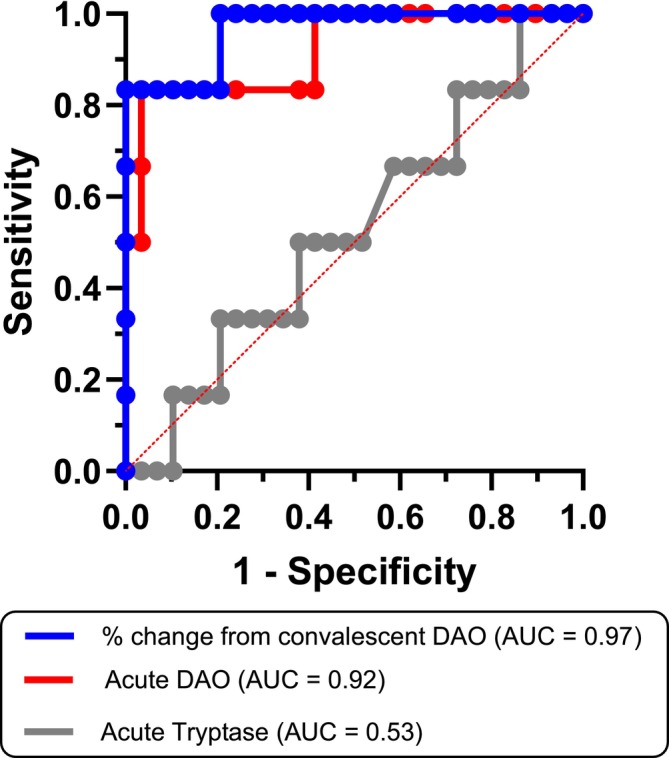
ROC curves comparing the discriminating performance of acute DAO and tryptase determination as well as the percentage change of DAO during acute episode compared to the convalescent sample.

With the gathered data, we determined the cut‐off values for the DAO concentration in the acute phase of anaphylaxis and the percentage increase of concentration compared to the convalescent sample to differentiate between the two subgroups of included patients. As indicated by the area under the ROC curve analysis, the absolute DAO concentration during episodes of anaphylaxis (AUC = 0.92, 95% CI 0.79–1.00) showed a high discriminating accuracy between *KIT* p.D816V‐positive and *KIT* p.D816V‐negative patients (Figure 5). Using a determined cut‐off of 53 U/mL (optimal cut‐off was determined based on the Youden index), the sensitivity and specificity of concentration in the acute phase are 83% and 97%, respectively. Hence, measuring DAO only during anaphylaxis and employing this cut‐off, 5 out of 6 patients would be correctly identified as *KIT* p.D816V‐positive; furthermore, 28 of 29 included patients would correctly reside in the *KIT* p.D816V‐negative subgroup (Figure 2A). Next, we assessed the usefulness of the change of DAO concentration compared to convalescent sampling in discriminating between *KIT* p.D816V‐positive and *KIT* p.D816V‐negative patients. Using a ROC curve analysis (AUC = 0.97, 95% CI 0.90–1.00), and based on the Youden index, we determined that the optimal cut‐off was a 223% increase in DAO concentration during an anaphylactic episode from convalescent, with sensitivity of 83% and specificity of 100% (Figure 5). In detail, *KIT* p.D816V‐positive patients were identified with the same success rate as with an absolute concentration threshold (5/6), whereas all of the 29 tested *KIT* p.D816V‐negative patients were correctly determined using the obtained threshold. On the other hand, acute tryptase determination was not able to discriminate between *KIT* p.D816V‐positive and *KIT* p.D816V‐negative patients (AUC = 0.53, 95% CI 0.28–0.77).

## 4 Discussion

In the emergency department study of patients with acute episodes of *Hymenoptera* venom, drug and food‐triggered anaphylaxis, we observed a marked acute rise in histamine‐degrading diamine oxidase during anaphylaxis only in patients with clonal mast cell disorder. All CMD patients had detectable *KIT* p.D816V variant in PBL, experienced severe HVA, and had normal BST levels. These findings indicate that massive DAO release during severe anaphylaxis is restricted to CMD and provide possible functional support for the reported association between *KIT* p.D816 and anaphylaxis severity. Measuring DAO during anaphylactic reactions may represent a valuable screening test for patients suspected of CMD. On the other hand, in the post‐mortem study, DAO showed limited specificity despite very high levels in fatal HVA with CMD, whereas post‐mortem tryptase proved a useful discriminating biomarker to support the diagnosis of fatal anaphylaxis in the presence of CMD.

The presence of underlying CMD is a strong predictor of reaction severity, particularly in patients with HVA [3, 4, 5, 6, 7, 8, 9]. A recent multicentric study corroborated this association, where nearly 40% of HVA patients with severe reactions harboured *KIT* p.D816V in their PBL, compared with approximately 10% with moderate reactions, and 1% with large local reactions, while it was absent in asymptomatic sensitised individuals [5]. The mechanisms driving greater severity in CMD remain incompletely defined and likely reflect the burden and intrinsic properties of *KIT* p.D816V‐positive MCs and potentially other *KIT* p.D816V‐positive myeloid cells (neutrophils, monocytes, eosinophils, and basophils) [10, 30]. How and if the massive release of DAO into the circulation during severe anaphylaxis in CMD contributes to clinical severity remains to be determined in future studies.

Our observation of increased release of DAO during anaphylaxis is consistent with animal data, although the physiological role of circulating DAO could not be unambiguously determined [21, 22], and complements the previous findings of DAO release into circulation in mastocytosis during severe reactions [23]. Crucially, we have clearly demonstrated for the first time that the acute DAO increase is restricted to CMD; *KIT* p.D816V‐positive patients exhibited substantially higher acute DAO and larger percentage changes from convalescence than *KIT* p.D816V‐negative patients. Currently, we do not know the pathophysiological mechanism leading to increased DAO. Potential mechanisms include heparin‐driven liberation of epithelial DAO, given that mast‐cell heparin is co‐stored with tryptase and, upon degranulation, can both displace DAO from intestinal epithelium [19, 23, 31] and activate FXIIa–bradykinin pathways that enhance vascular permeability, which might potentiate the severity of anaphylaxis [32]. Alternatively, DAO could be released from activated neutrophils [33], since neutrophil activation, demonstrated as upregulation of neutrophil gene signature [34] and other biomarkers associated with neutrophil activation [35], plays an important role in anaphylaxis. Although *KIT* p.D816V‐mutated neutrophils occur in CMD [30], their role in DAO release and clinical severity is unknown.

Clinically, measuring DAO during anaphylaxis may offer a simple, non‐invasive screen for occult underlaying CMD. In our cohort, patients with the *KIT* p.D816V variant in PBL had higher acute DAO concentrations and larger percentage increases from convalescence than *KIT* p.D816V‐negative patients. Acute DAO in *KIT* p.D816V‐positive cases was markedly elevated, also relative to recently published healthy adults [17] (median 101 vs. 18 U/mL; *p <* 0.0001), whereas acute DAO in *KIT* p.D816V‐negative patients mirrored healthy controls (median 14 vs. 18 U/mL; *p =* 0.905). Notably, none of the healthy controls exceeded 50 U/mL [17]. Receiver‐operating‐characteristic analyses identified two pragmatic criteria that discriminated CMD from non‐CMD: an acute DAO cut‐off of 53 U/mL and/or a ≥ 233% increase from the convalescent value. Using the proposed cutoff, DAO is a quick and affordable screening biomarker that, in conjunction with other tests, helps identify CMD in individuals with severe anaphylaxis. Tryptase concentration is usually measured at two different time points (acute and convalescent) [36], DAO dynamic measurement at the same time points could be beneficial in monitoring changes related to the acute state. In our data, DAO concentration changed significantly in a subgroup between acute and convalescent state; and a cut‐off of 233% increase from convalescent represents a highly meaningful diagnostic test used to distinguish between *KIT* D816V‐positive and *KIT* D816V‐negative patients. Our findings also align with previous reports [27, 36, 37], demonstrating that acute tryptase levels may rise comparably in patients with and without CMD. Together, these findings position DAO dynamics as a complementary tool to identify CMD in patients presenting with severe anaphylaxis, while mechanistic studies are needed to define causality and source. Notably, DAO screening would identify one additional CMD patient who would otherwise be missed when relying solely on the REMA score, a proposed scoring system for further CMD evaluation.

Notably, during acute anaphylaxis, DAO's functional capacity to degrade histamine is markedly reduced compared with pregnancy plasma, even when DAO concentrations are similarly high (including in *KIT* D816V‐positive individuals), consistent with prior observations in mastocytosis [23]. A pregnancy plasma sample (518 U/mL DAO) degraded exogenous histamine (100 ng/mL) almost completely within 60 min (~10 ng/mL), whereas acute anaphylaxis serum/plasma samples did not reduce histamine below 50 ng/mL after 60 min despite DAO levels up to 606 U/mL. This cannot be attributed to standard emergency medications, which do not inhibit DAO, and instead suggests an anaphylaxis‐associated mechanism of DAO inhibition or inactivation [23]. In clonal mast cell disorders, compromised histamine breakdown may help explain particularly severe reactions via prolonged histamine exposure. Although limited human material hampers mechanistic work, recombinant human DAO can efficiently degrade histamine in mastocytosis plasma and may overcome the inhibition or inactivation of endogenous DAO [38].

In the post‐mortem study, DAO has limited specificity; although levels were very high in fatal HVA with CMD, they were also elevated in several controls who died of cardiac arrest or injury/external causes. Prior work likewise shows poor diagnostic performance of post‐mortem DAO in anaphylaxis without any anamnestic data indicating mastocytosis [28], and our findings indicate this limitation extends to anaphylaxis with underlying CMD. By contrast, our data support post‐mortem tryptase as a useful discriminating biomarker for fatal anaphylaxis in the presence of CMD. Tryptase was markedly elevated in both fatal HVA cases with post‐mortem–identified CMD, while remaining low in controls who died of cardiac arrest or injury/external causes. Notably, both cases exceeded commonly cited post‐mortem cut‐offs for anaphylaxis (44–54 ng/mL) [28, 29, 39, 40], whereas all controls were below these thresholds. This aligns with recent reports that highlight tryptase as the most consistently informative post‐mortem biomarker and report diagnostic thresholds in this range (with 45 ng/mL showing strong discriminative capacity) [41]. However, because our series lacked fatal anaphylaxis cases without CMD, we cannot determine whether the magnitude of tryptase elevation is specific to anaphylaxis with CMD. Prior literature documents substantial tryptase increases in fatal anaphylaxis even in the absence of known mastocytosis, underscoring that elevated post‐mortem tryptase supports the diagnosis of fatal anaphylaxis irrespective of CMD status [28, 29, 39, 40, 41]. Best practice therefore relies on post‐mortem–specific (higher) tryptase thresholds rather than the clinical 11.4 ng/mL cut‐off, with interpretation mindful of confounders and of chronically elevated baselines in mast‐cell disorders. Overall, recent evidence converges on tryptase as the best‐validated post‐mortem marker, while alternative biomarkers remain limited and heterogeneous [28, 29, 39, 40, 41].

Our study has inherent limitations. The cohort was relatively small, reflecting the inherent difficulty of collecting paired samples during both the acute anaphylactic episode and convalescence. Another limitation is that we determined only DAO and tryptase in serum samples, and we did not determine other compounds, such as heparin. We also lacked functional experiments in isolated *KIT* p.D816V‐positive versus ‐negative cells to identify the cellular source of DAO and clarify how CMD modifies anaphylaxis severity. The predominant *Hymenoptera* venom trigger, potential confounding by medications administered during acute episodes, comorbidities, and a single‐center design may limit generalisability. In the post‐mortem analysis, variable intervals and conditions could have affected analyte stability. Larger, multicenter studies with standardised sampling and targeted mechanistic experiments are needed to validate and extend these findings.

In summary, according to our knowledge, we present the first evidence that pronounced DAO release during severe anaphylaxis is restricted to patients with CMD (*KIT* p.D816V‐positive patients). This finding offers initial functional insight into how *KIT* p.D816V‐positive cells may modulate anaphylaxis severity, although the precise mechanisms remain unclear. Clinically, measuring DAO during the acute episode is a simple, non‐invasive, and readily accessible test that might be helpful in screening for occult CMD among patients with severe anaphylaxis.

## Author Contributions

Matija Rijavec conceptualised the study, performed the experiments, analysed the data and wrote the manuscript. Žan Kogovšek performed the experiments and analysed the data. Jezerka Inkret, Peter Kopač and Mitja Košnik were involved in patient recruitment and clinical data. Peter Korošec supervised the study. All authors have been involved in revising the manuscript and have given final approval of the version to be published.

## Funding

This study was supported by the Slovenian Research and Innovation Agency (P3‐0360 and J3‐50114).

## Conflicts of Interest

The authors declare no conflicts of interest.

## Data Availability

The data that support the findings of this study are available from the corresponding author upon reasonable request.

## References

[1] L. L. Reber , J. D. Hernandez , and S. J. Galli , “The Pathophysiology of Anaphylaxis,” Journal of Allergy and Clinical Immunology 140 (2017): 335–348. 28780941 10.1016/j.jaci.2017.06.003 PMC5657389

[2] A. Muraro , M. Worm , C. Alviani , et al., “EAACI Guidelines: Anaphylaxis (2021 Update),” Allergy 77 (2022): 357–377. 34343358 10.1111/all.15032

[3] J. J. Lyons , J. Chovanec , M. P. O'Connell , et al., “Heritable Risk for Severe Anaphylaxis Associated With Increased α‐Tryptase–Encoding Germline Copy Number at TPSAB1,” Journal of Allergy and Clinical Immunology 147 (2021): 622–632. 32717252 10.1016/j.jaci.2020.06.035

[4] M. Kačar , M. Rijavec , J. Šelb , and P. Korošec , “Clonal Mast Cell Disorders and Hereditary α‐Tryptasemia as Risk Factors for Anaphylaxis,” Clinical and Experimental Allergy 53 (2023): 392–404. 36654513 10.1111/cea.14264

[5] P. Korošec , G. J. Sturm , J. J. Lyons , et al., “High Burden of Clonal Mast Cell Disorders and Hereditary α‐Tryptasemia in Patients Who Need Hymenoptera Venom Immunotherapy,” Allergy 79 (2024): 2458–2469. 38477502 10.1111/all.16084 PMC11939115

[6] P. Bonadonna and L. Scaffidi , “Hymenoptera Anaphylaxis as a Clonal Mast Cell Disorder,” Immunology and Allergy Clinics of North America 38 (2018): 455–468. 30007463 10.1016/j.iac.2018.04.010

[7] J. Stoevesandt , G. J. Sturm , P. Bonadonna , J. N. G. Oude Elberink , and A. Trautmann , “Risk Factors and Indicators of Severe Systemic Insect Sting Reactions,” Allergy 75 (2020): 535–545. 31194889 10.1111/all.13945

[8] J. Šelb , M. Rijavec , R. Eržen , et al., “Routine KIT p.D816V Screening Identifies Clonal Mast Cell Disease in Patients With Hymenoptera Allergy Regularly Missed Using Baseline Tryptase Levels Alone,” Journal of Allergy and Clinical Immunology 148 (2021): 621–626.e7. 33753098 10.1016/j.jaci.2021.02.043 PMC10964493

[9] P. Valent , K. Hartmann , J. Schwaab , et al., “Personalized Management Strategies in Mast Cell Disorders: ECNM‐AIM User's Guide for Daily Clinical Practice,” Journal of Allergy and Clinical Immunology. In Practice 10 (2022): 1999–2012.e6. 35342031 10.1016/j.jaip.2022.03.007

[10] T. Gülen and C. Akin , “Anaphylaxis and Mast Cell Disorders,” Immunology and Allergy Clinics of North America 42 (2022): 45–63. 34823750 10.1016/j.iac.2021.09.007

[11] J. J. Costa , G. D. Demetri , T. J. Harrist , et al., “Recombinant Human Stem Cell Factor (Kit Ligand) Promotes Human Mast Cell and Melanocyte Hyperplasia and Functional Activation In Vivo,” Journal of Experimental Medicine 183 (1996): 2681–2686. 8676090 10.1084/jem.183.6.2681 PMC2192599

[12] D. Terhorst‐Molawi , T. Hawro , E. Grekowitz , et al., “The Anti‐KIT Antibody, CDX‐0159, Reduces Mast Cell Numbers and Circulating Tryptase and Improves Disease Control in Patients With Chronic Inducible Urticaria (Cindu),” Journal of Allergy and Clinical Immunology 149 (2022): AB178.

[13] L. Maintz and N. Novak , “Histamine and Histamine Intolerance,” American Journal of Clinical Nutrition 85 (2007): 1185–1196. 17490952 10.1093/ajcn/85.5.1185

[14] O. Comas‐Basté , S. Sánchez‐Pérez , M. T. Veciana‐Nogués , M. Latorre‐Moratalla , and M. D. C. Vidal‐Carou , “Histamine Intolerance: The Current State of the Art,” Biomolecules 10 (2020): 1–26. 10.3390/biom10081181 PMC7463562 32824107

[15] M. C. J. Wolvekamp and R. W. F. De Bruin , “Diamine Oxidase: An Overview of Historical, Biochemical and Functional Aspects,” Digestive Diseases 12 (1994): 2–14. 8200121 10.1159/000171432

[16] M. Raithel , M. Küfner , P. Ulrich , and E. G. Hahn , “The Involvement of the Histamine Degradation Pathway by Diamine Oxidase in Manifest Gastrointestinal Allergies,” Inflammation Research 48, no. Suppl 1 (1999): S75–S76. 10350171 10.1007/s000110050414

[17] K. Arih , N. Đorđević , M. Košnik , and M. Rijavec , “Evaluation of Serum Diamine Oxidase as a Diagnostic Test for Histamine Intolerance,” Nutrients 15 (2023): 1–9. 10.3390/nu15194246 PMC10574399 37836530

[18] M. M. Refaat , A. S. Abdel‐Rehim , A. R. Elmahdi , N. A. Mohamed , and S. S. Ghonaim , “Diamine Oxidase Enzyme: A Novel Biomarker in Respiratory Allergy,” International Forum of Allergy & Rhinology 9 (2019): 1478–1484. 31532921 10.1002/alr.22426

[19] K. Toyoshima , S. Doi , T. Inoue , et al., “Plasma Diamine Oxidase Activity in Asthmatic Children,” Allergology International 45 (1996): 141–143.

[20] E. Vassilopoulou , G. N. Konstantinou , A. Dimitriou , Y. Manios , L. Koumbi , and N. G. Papadopoulos , “The Impact of Food Histamine Intake on Asthma Activity: A Pilot Study,” Nutrients 12 (2020): 1–13. 10.3390/nu12113402 PMC7694530 33167542

[21] F. Hahn , F. Pröhle , R. Mitze , and L. Degand , “Changes in the Histaminolytic Activity of the Guinea Pig Liver and Plasma During Anaphylaxis and After Heparin Injection,” International Archives of Allergy and Immunology 40 (1971): 340–350. 10.1159/000230417 4993764

[22] B. Rose and J. Leger , “Serum Histaminase During Rabbit Anaphylaxis,” Proceedings of the Society for Experimental Biology and Medicine (New York, N.Y.) 79 (1952): 379–381. 10.3181/00379727-79-19386 14920435

[23] T. Boehm , B. Reiter , R. Ristl , et al., “Massive Release of the Histamine‐Degrading Enzyme Diamine Oxidase During Severe Anaphylaxis in Mastocytosis Patients,” Allergy 74 (2019): 583–593. 30418682 10.1111/all.13663 PMC6590243

[24] M. Rijavec , J. Inkret , U. Bidovec‐Stojković , et al., “Fatal Hymenoptera Venom–Triggered Anaphylaxis in Patients With Unrecognized Clonal Mast Cell Disorder—Is Mastocytosis to Blame?,” International Journal of Molecular Sciences 24 (2023): 16368. 38003556 10.3390/ijms242216368 PMC10671356

[25] R. Vantur , M. Rihar , A. Koren , et al., “Chemokines During Anaphylaxis: The Importance of CCL2 and CCL2‐Dependent Chemotactic Activity for Basophils,” Clinical and Translational Allergy 10 (2020): 1–11. 33317619 10.1186/s13601-020-00367-2 PMC7737350

[26] J. D. Khoury , E. Solary , O. Abla , et al., “The 5th Edition of the World Health Organization Classification of Haematolymphoid Tumours: Myeloid and Histiocytic/Dendritic Neoplasms,” Leukemia 36 (2022): 1703–1719. 35732831 10.1038/s41375-022-01613-1 PMC9252913

[27] P. Valent , P. Bonadonna , K. Hartmann , et al., “Why the 20% + 2 Tryptase Formula Is a Diagnostic Gold Standard for Severe Systemic Mast Cell Activation and Mast Cell Activation Syndrome,” International Archives of Allergy and Immunology 180 (2019): 44–51. 31256161 10.1159/000501079 PMC7115850

[28] D. E. Mayer , A. Krauskopf , W. Hemmer , K. Moritz , R. Jarisch , and C. Reiter , “Usefulness of Post Mortem Determination of Serum Tryptase, Histamine and Diamine Oxidase in the Diagnosis of Fatal Anaphylaxis,” Forensic Science International 212 (2011): 96–101. 21664082 10.1016/j.forsciint.2011.05.020

[29] E. Edston , O. Eriksson , and M. Van Hage , “Mast Cell Tryptase in Postmortem Serum‐Reference Values and Confounders,” International Journal of Legal Medicine 121 (2007): 275–280. 16710735 10.1007/s00414-006-0101-2

[30] P. Navarro‐Navarro , I. Álvarez‐Twose , A. Pérez‐Pons , et al., “KITD816V Mutation in Blood for the Diagnostic Screening of Systemic Mastocytosis and Mast Cell Activation Syndromes,” Allergy 78 (2023): 1347–1359. 36385619 10.1111/all.15584

[31] S. C. Alter , D. D. Metcalfe , T. R. Bradford , and L. B. Schwartz , “Regulation of Human Mast Cell Tryptase. Effects of Enzyme Concentration, Ionic Strength and the Structure and Negative Charge Density of Polysaccharides,” Biochemical Journal 248 (1987): 821–827. 2449172 10.1042/bj2480821 PMC1148623

[32] A. Sala‐Cunill , J. Björkqvist , R. Senter , et al., “Plasma Contact System Activation Drives Anaphylaxis in Severe Mast Cell‐Mediated Allergic Reactions,” Journal of Allergy and Clinical Immunology 135 (2015): 1031–1043.e6. 25240785 10.1016/j.jaci.2014.07.057

[33] T. Boehm , M. Karer , P. Matzneller , et al., “Human Diamine Oxidase Is Readily Released From Activated Neutrophils Ex Vivo and In Vivo but Is Rarely Elevated in Bacteremic Patients,” International Journal of Immunopathology and Pharmacology 34 (2020): 2058738420954945. 32997559 10.1177/2058738420954945 PMC7533923

[34] M. Rijavec , A. Maver , P. J. Turner , et al., “Integrative Transcriptomic Analysis in Human and Mouse Model of Anaphylaxis Identifies Gene Signatures Associated With Cell Movement, Migration and Neuroinflammatory Signalling,” Frontiers in Immunology 13 (2022): 1–15. 10.3389/fimmu.2022.1016165 PMC9772259 36569939

[35] A. Francis , E. Bosio , S. F. Stone , et al., “Neutrophil Activation During Acute Human Anaphylaxis: Analysis of MPO and sCD62L,” Clinical and Experimental Allergy 47 (2017): 361–370. 27906487 10.1111/cea.12868

[36] P. Valent , C. Akin , and M. Arock , “Reversible Elevation of Tryptase Over the Individual's Baseline: Why Is It the Best Biomarker for Severe Systemic Mast Cell Activation and MCAS?,” Current Allergy and Asthma Reports 24 (2024): 133–141. 38308674 10.1007/s11882-024-01124-2 PMC10960756

[37] M. Beyens , A. Toscano , D. Ebo , T. Gülen , and V. Sabato , “Diagnostic Significance of Tryptase for Suspected Mast Cell Disorders,” Diagnostics (Basel, Switzerland) 13 (2023): 3662. 38132246 10.3390/diagnostics13243662 PMC10742504

[38] E. Gludovacz , K. Schuetzenberger , M. Resch , et al., “Heparin‐Binding Motif Mutations of Human Diamine Oxidase Allow the Development of a First‐In‐Class Histamine‐Degrading Biopharmaceutical,” eLife 10 (2021): e68542. 34477104 10.7554/eLife.68542 PMC8445614

[39] R. Tse , C. X. Wong , K. Kesha , et al., “Post Mortem Tryptase Cut‐Off Level for Anaphylactic Death,” Forensic Science International 284 (2018): 5–8. 29331682 10.1016/j.forsciint.2017.12.035

[40] J. Garland , B. Ondruschka , U. Da Broi , C. Palmiere , and R. Tse , “Post Mortem Tryptase: A Review of Literature on Its Use, Sampling and Interpretation in the Investigation of Fatal Anaphylaxis,” Forensic Science International 314 (2020): 110415. 32717658 10.1016/j.forsciint.2020.110415

[41] B. Karthikeyan , R. I. James , J. Daniel , et al., “Utility of Biomarkers in the Postmortem Diagnosis of Fatal Anaphylaxis: A Scoping Review,” Legal Medicine (Tokyo, Japan) 74 (2025): 102610. 40163933 10.1016/j.legalmed.2025.102610

